# Recent Advances in CRISPR/Cas9-Based Genome Editing Tools for Cardiac Diseases

**DOI:** 10.3390/ijms222010985

**Published:** 2021-10-12

**Authors:** Juliët Schreurs, Claudia Sacchetto, Robin M. W. Colpaert, Libero Vitiello, Alessandra Rampazzo, Martina Calore

**Affiliations:** 1 Department of Molecular Genetics, Faculty of Health, Medicine and Life Sciences, Faculty of Science and Engineering, Maastricht University, ER 6229 Maastricht, The Netherlands; juliet.schreurs@gmail.com (J.S.); c.sacchetto@maastrichtuniversity.nl (C.S.); R.Colpaert@maastrichtuniversity.nl (R.M.W.C.); 2 Department of Biology, University of Padova, 35131 Padova, Italy; libero.vitiello@bio.unipd.it (L.V.); alessandra.rampazzo@unipd.it (A.R.)

**Keywords:** genome editing, CRISPR/Cas9, base editing, prime editing, cardiac disease

## Abstract

In the past two decades, genome editing has proven its value as a powerful tool for modeling or even treating numerous diseases. After the development of protein-guided systems such as zinc finger nucleases (ZFNs) and transcription activator-like effector nucleases (TALENs), which for the first time made DNA editing an actual possibility, the advent of RNA-guided techniques has brought about an epochal change. Based on a bacterial anti-phage system, the CRISPR/Cas9 approach has provided a flexible and adaptable DNA-editing system that has been able to overcome several limitations associated with earlier methods, rapidly becoming the most common tool for both disease modeling and therapeutic studies. More recently, two novel CRISPR/Cas9-derived tools, namely base editing and prime editing, have further widened the range and accuracy of achievable genomic modifications. This review aims to provide an overview of the most recent developments in the genome-editing field and their applications in biomedical research, with a particular focus on models for the study and treatment of cardiac diseases.

## 1. Introduction

Genome editing is a catch-all definition that refers to a variety of techniques capable of achieving site-specific DNA modifications in a living cell. The concept itself is far from new, as the first reports of targeted genetic changes date back to the late 1970s and the 1980s [1,2]. The early development of this field was made possible by important findings related to the cellular mechanisms involved in genome plasticity, one of which is homologous recombination (HR), a process that can allow the integration of an exogenous DNA segment into the host genome [3]. Shortly after, it became apparent that this process could be greatly enhanced through the induction of a double-strand break (DSB) within the target region of the DNA [4], which is the foundational concept in modern genome editing techniques [5]. To generate a site-specific DSB, various endonuclease-based methods have been developed, capable of cutting the target DNA with high site-specificity. Such cleaving can then trigger the DNA repair pathways of the target cell: the homology-directed repair (HDR) and the non-homologous end-joining (NHEJ) mechanism [6].

**Figure 1:**
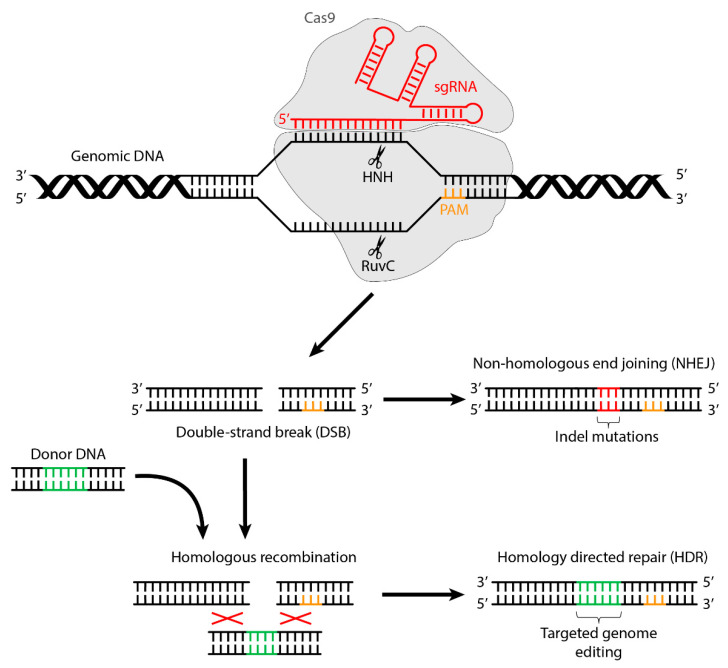
Mechanism of genome editing by CRISPR/Cas9.

A crucial breakthrough in genome editing occurred with the discovery of the CRISPR/Cas9 system in bacteria, in which it serves as a defense system against bacteriophagic infections. The CRISPR acronym derives from “clustered regularly interspaced short palindromic repeats”, while Cas derives from “CRISPR-associated endonuclease”. Since the discovery of the biological significance of the mechanisms behind it, and thanks to its simplicity and adaptability [7], CRISPR/Cas9 has ushered in the current booming era of genome editing in a multitude of fields, both in vitro and in vivo, including the study of gene function, disease modeling, and the testing of new therapeutic approaches [8]. Numerous CRISPR/Cas systems are known today (e.g., Cas9, Cas12a), classified on the basis of the number of effectors, types, and subtypes; still, the one most frequently used remains Cas9 [9]. The system relies on simple Watson-Crick base pairing rules between a specifically designed RNA and the target DNA sequence. It employs CRISPR RNA (crRNA) as a guide in a complex with CRISPR-associated (Cas) proteins due to the presence of the variable “protospacer” sequence [10,11]. A second trans-activating CRISPR RNA (tracrRNA)—a small trans-encoded noncoding RNA—is necessary for triggering pre-crRNA processing by RNase III and the subsequent activation of DNA cleavage [12]. Each crRNA hybridizes with one of these tracrRNAs to create a tracrRNA:crRNA duplex, also referred to as single-guide RNA (sgRNA), which directs the Cas9 protein—a large multifunctional protein with two nuclease domains, HNH and RuvC-like—to the target DNA sequence for subsequent cleavage (Figure 1). This dual RNA holds two essential features: a 20-nucleotide sequence corresponding to the protospacer sequence localized at the 5′ end of the sgRNA, which directs the Cas9 to the DNA target site; and a double-stranded structure at the 3′ end that binds to this cleavage protein [11]. Once Cas9 binds to its sgRNA, the complex is able to search for the complementary target DNA site [13]. Target recognition and subsequent cleavage will only occur when the target sequence is adjacent to a short “seed” sequence known as a “protospacer adjacent motif” (PAM) (Figure 1), which differs based on the Cas enzyme used. For example, *Streptococcus pyogenes* Cas9 (SpCas9) recognizes the 3′NGG sequence, while *Staphylococcus aureus* Cas9 (SaCas9) recognizes either 3′NNGRRT or NNGRR(N) [14]. After cleavage, the nuclease-induced DSB can be repaired by NHEJ or HDR, which are functional in nearly all cell types. NHEJ can generate an insertion or deletion (indel), thereby disrupting the translational reading frame. On the other hand, HDR is able to introduce a specific point mutation or insert the desired sequence with the use of a donor template in dividing cells (Figure 1) [12].

Importantly, upon modifications of Cas9—leading to the so-called dead Cas9, or dCas9—the system has also been used to insert methyl groups and block or activate transcription in precise positions [15]. As far as dCas9 applications for genome editing are concerned, a clear example is represented by base editing. DNA base editors, together with prime editors, belong to a new generation of CRISPR genome editing tools that overcome the main limitations associated with CRISPR/Cas9 systems, such as low editing efficiency and a moderate risk of off-target effects.

### A New Generation of Genome Editing Tools: Base Editing and Prime Editing

The two classes of DNA base editors described are cytosine base editors (CBEs) and adenine base editors (ABEs), which, collectively, are able to induce C > T, T > C, A > G, and G > A transitions [16] without the need to introduce a DSB or to provide an additional exogenous template. With higher editing efficiency and lower risk of off-target effects, both base editors represent an important development in genome editing technologies and are potentially able to correct the most common type of pathogenic single-nucleotide variations accounting for different human pathogenic conditions [17,18].

**Figure 2:**
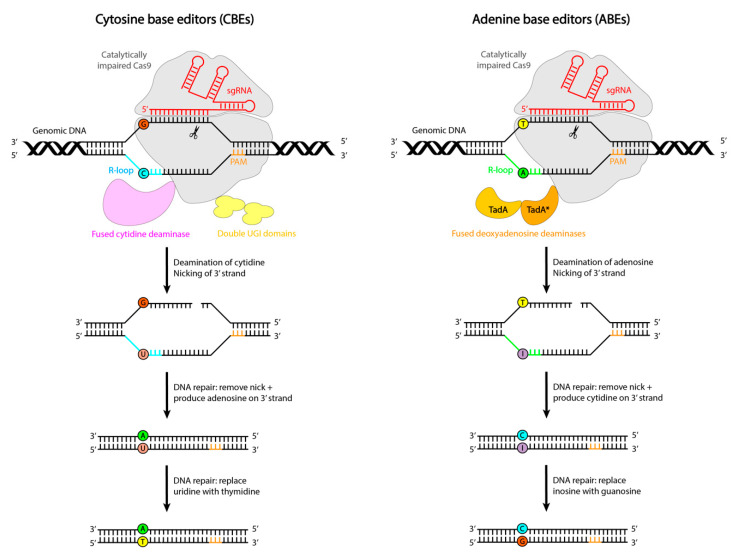
Mechanisms of installing transition point mutations with base editors. (**Left**) Cytosine base editor and (**Right**) adenine base editor.

CBEs consist of a macromolecular complex in which a catalytically impaired CRISPR/Cas9 nuclease is fused to an APOBEC1 deaminase enzyme and a uracyl glycosylase inhibitor protein. Upon Cas binding, hybridization of the guide RNA to its target DNA sequence leads to the displacement of the PAM-containing DNA strand to create an R-loop by local denaturation of the DNA duplex (Figure 2). In order to convert cytosines within the R-loop to uracils, which are read as thymines by polymerases, CBEs employ a cytidine deaminase, APOBEC1 (Figure 2, left). On the other hand, for the conversion of adenosines within the R-loop to inosines, which are read by polymerases as guanines, ABEs utilize a tRNA adenosine deaminase enzyme: laboratory-evolved TadA* deoxyadenosine deaminases (Figure 2, right) [19]. Deamination within this editing window initially produces uridine and inosine, thereby creating a mismatched DNA base pair with the base on the opposite strand, enabling A to G conversions (Figure 2) [17].

However, these uracil and inosine intermediates are mutagenic, and DNA repair mechanisms have evolved to remove these bases from the DNA. For uracil, this is done by uracil DNA N-glycosylase (UNG2). To increase the half-life of uracil and consequently increase the purity—referred to as the ratio of intended edits to random edits—and editing efficiency, CBEs are fused to uracil glycosylase inhibitor proteins (UGIs) (Figure 2, left). 

Further improvements to editing efficiency were achieved by designing the Cas nuclease in such a manner that it generates a nick in the non-edited DNA strand, thereby stimulating cellular repair of the non-edited strand using the edited strand as a template [18]. Additional advancements were made with the replacement of wild-type SpCas with its other variants, linker optimization, and the injection of a second fragment of UGI [20]. 

With these mechanisms, base editors can introduce point mutations more efficiently and with fewer undesired byproducts compared to the CRISPR/Cas9 method [17].

Whereas base editing lacks the capability to perform a transversion mutation other than C > T, T > C, A > G, and G > A without excess byproducts, prime editing is able to efficiently mediate targeted insertions, deletions, all 12 possible point mutations, and combinations thereof. With this recently developed method, the desired genetic information is directly written into a specified DNA site without requiring DSBs or a donor DNA template [19]. Prime editing considerably expands the capabilities and scope of genome editing, potentially being able to correct up to 89% of human disease-causing genetic variants [21]. 

**Figure 3:**
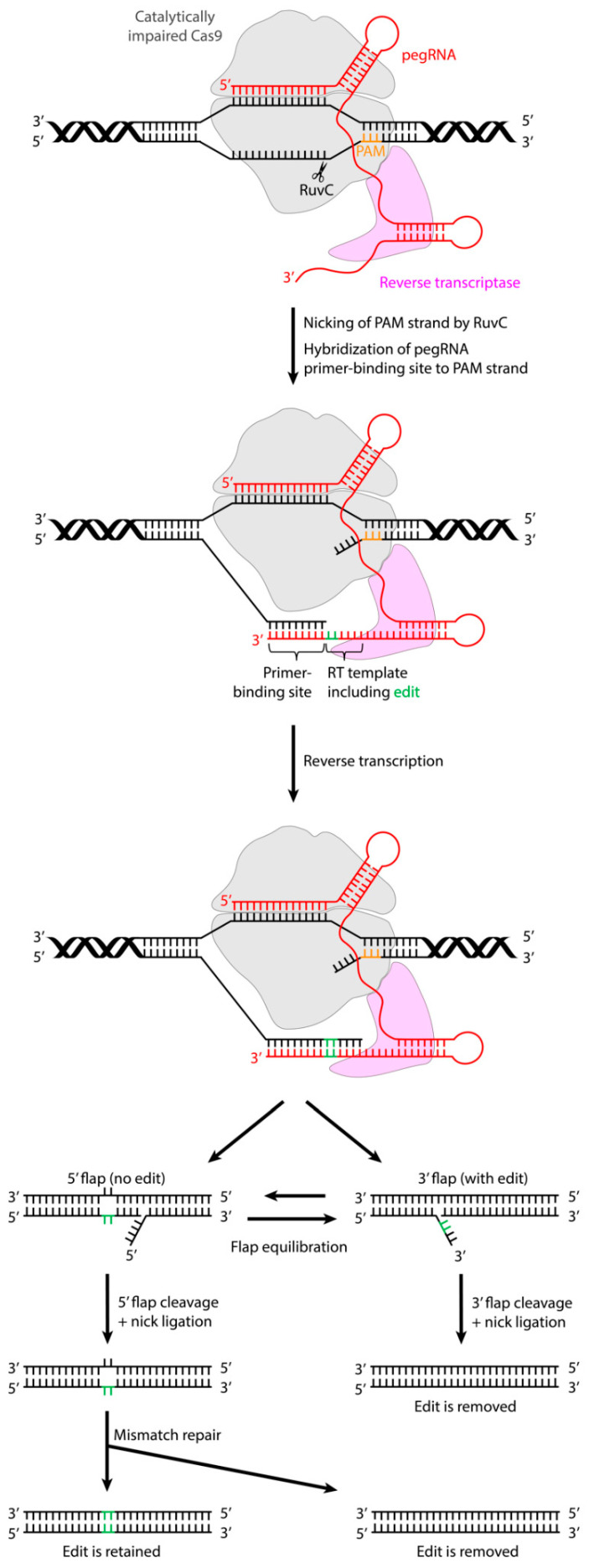
Mechanism of the action of the prime editing complex.

Prime editors can be created by fusing a catalytically impaired Cas9 endonuclease (Cas9 nickase), with an inactivated HNH nuclease domain, to an engineered reverse transcriptase (RT) domain (Figure 3). The targeting of these prime editor proteins to the editing site is carried out by the prime editing guide RNA (pegRNA) (Figure 3), which defines the target DNA site in its spacer region and contains three main elements: an RT template encoding the desired edit, a region of homology to the target site to guide the synthesis of the edited DNA strand by DNA repair, and a primer binding site (PBS) that allows the 3′ end of the nicked DNA strand to hybridize with the pegRNA, thereby initiating RT activity [19,22].

Various prime editor systems have been developed by Anzalone et al., starting from PE1, in which Cas9 nickase is fused to a wild-type Moloney murine leukemia virus (M-MLV) RT [21]. To increase the editing efficiency, this RT domain was substituted with an engineered pentamutant M-MLV RT in PE2. Further advancements were made with PE3, which combines the PE2 fusion protein and pegRNA with an additional sgRNA that nicks the non-edited strand, thereby further increasing the editing efficiency. To reduce the risk of indels and DSBs, PE3b was designed to contain a sgRNA with spacers matching the edited strand, but not the original allele. Using this strategy, the non-edited strand is only nicked after the PAM strand has been converted to the desired sequence.

Upon target binding by the pegRNA, the Cas9 RuvC nuclease domain nicks the PAM-containing DNA strand and liberates the 3′ end at the target DNA site (Figure 3). The PBS sequence included in the 3′ pegRNA extension can hybridize with the 3′ end of the nicked target DNA strand, thus forming a primer–template complex. This is necessary for the RT domain to synthesize the edited DNA strand, based on the sequence of the pegRNA RT template, onto the 3′ end of the target DNA strand (Figure 3). After reverse transcription, the newly synthesized DNA strand comprising the edit exists as a 3′ DNA flap that is in equilibrium with a 5′ flap which still contains the unedited DNA sequence. Cellular DNA repair processes are thought to cleave the unedited 5′ flap, which allows the edited 3′ flap to be incorporated into the target DNA site, generating a heteroduplex DNA consisting of one edited and one non-edited strand (Figure 3). Upon cleavage of the edited 3′ flap and subsequent ligation and DNA repair, an unedited DNA duplex is formed (Figure 3). To achieve the permanent installation of the desired edit, DNA repair of the non-edited strand must occur. This can be promoted by the use of an additional sgRNA directing a nick in the non-edited strand, thereby stimulating the resynthesis of this strand using the edited strand as a template, such that a fully edited DNA duplex is formed [19].

## 2. Applications of CRISPR/Cas9-Based Genome Editing Systems in Cardiac Disease Models

Cardiovascular disorders are the main cause of death worldwide [23]; therefore, there is an urgent need to improve existing knowledge of disease mechanisms and to develop more efficient therapies. Genome editors represent a very valuable tool for the introduction or correction of a site-specific mutation, both in vitro and in vivo, both to model these diseases as well as to be tested as a novel therapeutic approach. However, given that HDR is restricted to the S and G2 phases of the cell cycle, post-mitotic tissues, such as the heart, give rise to an additional challenge for CRISPR/Cas9 genome editing, as HDR is typically very inefficient in these tissue types [24]. Therefore, in this review, special attention will be paid to these disorders, rendering relevant insights on the use and potential further development of the most recently developed editing methods in cardiac research. 

### 2.1. In Vitro Models

In recent years, advances in the technology for the generation of human-induced pluripotent stem cell (hiPSC)-derived cardiomyocytes (hiPSC-CMs) from affected patients opened up new possibilities for the modeling of cardiac diseases [25]. In this context, it is crucial to choose the proper control to assess the influence of a given mutation on the observed phenotype. Many studies on hiPSCs use age-matched unaffected cells as a control [26,27,28], which have the significant limitation of carrying differences in genetic background and other confounders. A solution to this problem is the use of isogenic cell lines, which have an identical genetic background and can match culture conditions, origin, epigenetics, and differentiation capacity. This makes isogenic cells the optimal control for interpreting experimental data as a consequence of the mutation of interest, without background bias. Genome-editing tools such as CRISPR/Cas9 have already been broadly used to generate isogenic cell lines by either correcting the mutation in patient-specific cells or by introducing the mutation into unaffected cells (for recent reviews, see Vermersch et al. [8] and My and Di Pasquale [29]). In both cases, several technical aspects have to be taken into account. Cas9 endonucleases have shown broad variation in their activity, even when the sgRNAs are designed in close genomic proximity. For this reason, it is often necessary to test more than one sgRNA to target different DNA sequences around the editing site. The delivery of CRISPR/Cas9 to hiPSCs may also be challenging with common transfection reagents; to date, nucleofection is the most robust delivery method. Another important aspect to consider is single-cell cloning survival. Indeed, the inefficiency in editing hiPSCs is largely due to low cell viability after manipulation. Following nucleofection, hiPSCs are plated at a single-cell density, through limiting dilution or single-cell sorting, which normally leads to the loss of a part of the transfected cells, thus reducing the probability of identifying edited cell clones. However, in recent years, most of the above-mentioned conditions have been optimized and standardized, promoting the efficient use of CRISPR/Cas9 technology for the generation of hiPSC isogenic lines. The use of these approaches, alone or combined, led to the identification of novel aspects of the mechanistic events associated with hypertrophic cardiomyopathy, PRKAG2 cardiac syndrome, dilated cardiomyopathy, arrhythmogenic cardiomyopathy, Noonan syndrome, and others [30,31,32,33,34].

Moreover, CRISPR/Cas9 has been broadly used to generate in vitro allele-specific knockout models of cardiac diseases, such as the channelopathy long QT syndrome (LQTS) [35]. Cardiac diseases are often associated with electrical abnormalities and hiPSC-CMs recapitulate these alterations [36]. Similarly, cardiac diseases are often linked to mechanical alterations. The established methods that are currently available to measure mechanical force (e.g., optical cell stretcher [37], optical flow-based displacement analysis [38], and atomic force microscopy [39]), in combination with genome editing techniques for the generation of novel disease models, may lead to a better understanding of the mechanical phenotypes observed in vitro, shedding light on the stress–strain relationship and other useful readouts.

Therefore, these studies not only shed light on the pathophysiological mechanisms of cardiac diseases but also offered important insights into potential therapeutic approaches for hereditary cardiac diseases. Interestingly, CRISPR/Cas9 was also used to perform genome-scale functional screening. Sapp et al. successfully investigated the mechanisms underlying doxorubicin-mediated cell death in hiPSC-CMs [40].

On the other hand, only recently were base editing and prime editing applied to rescue phenotypes in vitro in models of cardiac diseases. Chemello et al. employed both base editing and prime editing to restore dystrophin protein expression in iPSC-CMs by inducing exon skipping or exon reframing, respectively, for the correction of the *DMD* exon 51 deletion mutation [41]. This deletion leads to a premature stop codon in exon 52 and, ultimately, a truncated, non-functional dystrophin protein. In this study, to generate a human iPSC-CM model, an isogenic hiPSC line was created with CRISPR/Cas9 carrying an exon 51 deletion in the *DMD* gene. Subsequent base editing on the mutant hiPSCs, before the differentiation, rendered a restored expression of the dystrophin protein in hiPS-CMs [41]. 

Additionally, prime editing was employed as an alternative correction method. This system was utilized via nucleofection to reframe the correct open reading frame of exon 52 and also showed the efficient correction and restoration of dystrophin expression in hiPSC-CMs [41].

Together, these results demonstrate the application and effectiveness of base editing and prime editing for the correction of several *DMD* mutations and open intriguing new opportunities for the study and possibly treatment of other genetic cardiac diseases.

### 2.2. In Vivo Models

Genome editing has also been used in vivo to generate disease models and to test novel therapeutic approaches for diseases such as cardiac disorders. Up to now, CRISPR/Cas9 is the genome-editing tool that is most frequently used for the generation of in vivo models of cardiac disorders. Meanwhile, base editing and prime editing are still in their infancy, despite some in vivo studies that have already been performed [42,43]. 

When using genome editing to correct a mutation that leads to an autosomal dominant disorder, it is essential to disrupt solely the mutant allele while keeping the wild-type allele intact [44]. This was accomplished in a study employing CRISPR/Cas9, aiming to correct a dominant mutation in the *PRKAG2* gene leading to PRKAG2 cardiac syndrome, characterized by ventricular tachyarrhythmia and progressive heart failure [44]. Post-natal transgenic and knock-in mice received a single injection of Cas9/sgRNA packaged into an AAV9 vector. As a result of this in vivo editing, left ventricular wall thickness was significantly reduced and ECG abnormalities were normalized. These findings highlight the potential of CRISPR/Cas9 to serve as a therapeutic approach to treat PRKAG2 cardiac syndrome as well as other dominant genetic cardiac diseases [44]. 

CRISPR/Cas9 was also used to correct large deletions in vivo, such as those responsible for Duchenne muscular dystrophy (DMD). El Refaey et al. targeted exon 23 of the dystrophin gene by using SaCas9—for its smaller size—and sgRNA packaged into an AAVrh74 and delivered these to neonatal mice [45]. The results showed a partial rehabilitation of the expression of the dystrophin protein in cardiomyocytes, as well as the structural restoration of the cardiac myofiber, together with a decreased fibrotic area in the heart. Additionally, a functional improvement of contractility was found in AAV-treated mice, indicating that the degree of cardiac phenotype rescue exceeded the efficiency of gene repair, thus suggesting that incomplete genome editing efficiency could still provide relevant clinical benefits. These findings demonstrate the therapeutic potential of in vivo editing by CRISPR/Cas9 for the functional and structural rehabilitation of cardiomyopathy associated with DMD [45].

More recently, base editing has also been employed as a genome editing approach to treat DMD in mice. Xu and colleagues succeeded in correcting the DMD-causing mutation in adult mdx4cv mice treated with a modified ABE (iABE-NGA), characterized by an engineered NG PAM-interacting domain variant that requires a more relaxed PAM sequence nearby the mutation site, besides showing improved on-target DNA editing activity and specificity [46]. After the systemic AAV9-mediated delivery of iABE-NGA, the treated mdx4cv mice showed an average of 32.6 ± 2.0% T-to-C editing at 10 weeks of age, together with a significant increase in the number of dystrophin-positive cardiomyocytes (41.9 ± 10.5%), consistent with the increased level of proteins in the heart. Interestingly, the authors also investigated the long-term impact of this approach, observing a near-complete dystrophin restoration (95.9 ± 1.6%) in the hearts of the treated mdx4cv mice at 10 months of age, which is explained by an average of 84.6 ± 2.6% T-to-C editing [46].

An outstanding study recently demonstrated the potential of base editing for the correction of disease-causing mutations in utero, thus preventing the onset of certain pathologies [47]. The AAV9-mediated in utero intravascular delivery of an ABE successfully corrected a nonsense *Idua* gene mutation in a mouse model of mucopolysaccharidosis type I (MPS-IH), a lysosomal storage disease affecting multiple organs, including the heart. In utero-treated mice showed a reduced cardiac lysosomal accumulation of glycosaminoglycans, increased IDUA activity in the heart, and improved echocardiographic function compared to control mice as a result of partial cardiomyocyte editing (13.9 ± 0.8%). Considering the natural characteristics of the developing fetus, as well as the timing of the onset of some diseases, prenatal base editing might become an attractive therapeutic approach, and this study confirms the potential of this system. Additionally, the authors assessed the post-natal effect of the ABE treatment, confirming the attenuation of the disease progression in the treated mice at 10 weeks of age [47]. 

One of the main issues with genome editors in vivo concerns their delivery to the target tissue, due to the large size of the key components and the low capacity of the delivery vectors. To circumvent this, K. J. Carroll et al. created a transgenic mouse model that constitutively expressed high levels of Cas9 exclusively in the heart, thereby only requiring AAV9 to deliver sgRNA, which is within its packing limit [48]. This was achieved by targeting myosin heavy chain 6 (*Myh6*) gene expression with cardiotropic AAV9 to deliver the associated sgRNA. The described method, cardioediting, is valuable for disease modeling, can provide a manner for the rapid editing of genes in the heart, and can be used to explore possible gene therapies regarding cardiac disease and dysfunction [48]. However, the mosaic pattern of gene disruption is an important factor to take into consideration when employing this method [49].

As an alternative approach, many research groups use a dual-vector system to deliver genome editing components separately in AAV constructs, allowing for the modification of the ratio between the two components, which proved to be vital for efficient targeting [50]. Recently, Chemello et al. used base editing to restore dystrophin protein expression in a DMD mouse model by inducing exon skipping for the correction of the *DMD* exon 51 deletion mutation [41]. This research group utilized an optimized ABE to induce a single nucleotide transition in *DMD* exon 50 of a mouse model harboring a deletion of exon 51. This system was packaged into AAV9 with the use of a split-intein system, resulting in the successful skipping of exon 50 and yielding the restoration of functional dystrophin expression [41]. This study depicts the potential of the base editing system, foretokening future in vivo studies using base editors and potentially prime editors in the heart. 

Another recent study reported by Koblan et al. assessed in vivo base editing by employing an ABE to correct the mutation in the lamin A (*LMNA*) gene associated with Hutchinson-Gilford progeria syndrome (HGPS) [51]. This mutation causes the mis-splicing of RNA, leading to the production of progerin, a toxic protein that accelerates aging in children and leads to early death, commonly caused by cardiovascular disease. The ABE was utilized in fibroblasts derived from children with this syndrome as well as in a mouse model carrying the human *LMNA* mutation. A dual AAV9 vector system was used to transduce ABE into progeria-relevant tissues, including the heart and muscle of a mouse model carrying the human *LMNA* mutation. A persistent correction of 20–60% was achieved, giving rise to the restoration of normal splicing and diminished levels of progerin, as well as notably improving vascular disease, thereby increasing the lifespan of ABE-treated mice by 2.4-fold compared to the control group [51]. These outcomes highlight the therapeutic potential of base editors for treating HGPS and other genetic disorders.

## 3. Limitations of Genome Editors

The different genome editing systems vary in terms of efficiency, off-target effects, and sensitivity. The therapeutic applications of genome editing remain restricted by technical and biological problems. Before utilizing a particular technique, several technical and ethical considerations need to be addressed, especially for gene therapy. The identified hurdles yet to be overcome, such as off-target effects, the efficacy of HDR, the fitness of edited cells, immunogenicity, as well as efficiency and specificity, give rise to future directions for the optimization of genome editing.

### 3.1. Off-Targets

One of the major concerns for the application of CRISPR/Cas9 is the high frequency of off-targets in human cells, observed in varying cell types, including cardiomyocytes [52,53]. Off-targets result from the nonspecific activity of Cas9 at nontarget sites as a consequence of faulty sgRNA binding. These unwanted mutations most likely occur at locations in the genome that possess a similar sequence to the target site. Off-target modifications can be predicted by a number of accessible computational programs, such as Cas-OFFinder (http://www.rgenome.net/cas-offinder/) [54], CCTop (https://cctop.cos.uni-heidelberg.de:8043/index.html) [55], and CRISPOR (http://crispor.tefor.net) [56]. However, it has been shown that many of the off-targets identified in cells after genome editing were not included among the off-target sites predicted by these programs [57], suggesting the inadequate predictive power of these computational tools and making it hard to anticipate exactly where off-targets might occur as well as their prevalence. On the other hand, unwanted edits can be efficiently monitored with the use of whole-genome sequencing [58]. Several attempts to reduce off-target effects have been carried out. Some groups achieved this by altering the binding site of Cas9 or by utilizing paired Cas9 nickases as an alternative to a sole Cas9 protein. Both approaches demonstrated diminished off-target binding and subsequent cleavage [52]. Additionally, the optimization of guide designs and engineered Cas9 variants have also been proven to minimize the frequency of off-target events [53]. Although off-target events may be rare with these improvements, they must still not be underestimated, as, in principle, with a mutation of the wrong gene in just a single cell, a damaging and possibly fatal complication could occur.

Besides the potentially dangerous effects of off-targets, on-target mutagenesis should also be taken into account. As opposed to the desired on-target edits, the introduction of a specific modification at the target site with HDR after the generation of a DSB by CRISPR/Cas9 can be inefficient. Particularly in post-mitotic tissues, NHEJ can lead to disruption of the target gene, resulting in potentially more disrupted cells than cells containing the desired edit. This limitation is particularly problematic in the case of the correction of a disease-causing mutation in vivo and should be taken into account before employing a technique such as CRISPR/Cas9 [58]. 

Base editors can also potentially induce off-target editing, both outside and within the activity window [18]. In the latter case, any cytidine or adenine can be modified by the corresponding deaminase domain of the base editor, resulting in bystander off-target modifications. To reduce the possibility of bystander off-targets, a narrow editing window is preferred. Recent studies indicate that this type of off-target effect can be controlled by tuning the activity of the deaminase in the editor enzyme, without disrupting the efficiency of the on-target DNA base editing [59,60]. Moreover, base editors render a low but detectable rate of indel formation, especially CBEs, which generate more unanticipated edits compared to ABEs, reducing product purity [61]. As a consequence of the weaker capability of cells to excise inosine from DNA than uracil, ABEs display a very high product purity (≥99.9%) with a negligible indel rate (≤0.1%), whereas CBEs show an editing efficiency of 37% and 1.1% indel generation [61]. This current flaw of CBEs reduces its employability and may be alleviated through optimization efforts.

Lastly, prime editing seems to be associated with lower off-target mutagenesis in comparison to base editing and CRISPR/Cas9 [21], which is a result of the fact that prime editing has a lower tolerance for mismatches in various regions of the pegRNA, such as the RT template and PBS, that serve as additional checkpoints alongside the spacer sequence [62]. However, as a consequence of the RT extension included in the pegRNA, although occurring at low frequency, reverse transcription of 3′-extended pegRNAs could potentially proceed into the guide RNA scaffold, resulting in small scaffold sequence insertions which contribute to indels at the target site [16].

### 3.2. Flexibility of the PAM Sequence

Another major restriction of CRISPR/Cas9 is the requirement for a PAM sequence in proximity to the target site, limiting its applicability and effectiveness. In fact, it has been shown that the efficiency of editing rapidly decreases with an increase in distance from the cut site [63]. To overcome this issue, several Cas9 orthologs that recognize different PAM sequences can be used, thereby expanding the genomic target window of CRISPR/Cas9. However, while some variants have a broader PAM recognition, they have an increased size relative to the most commonly used SpCas9. This complicates delivery and hinders its editing efficiency [64], thereby reducing its potential application. SaCas9 is a smaller ortholog, making it easier to package into AAV vectors. On the other hand, this variant has a longer PAM sequence, hence narrowing the target window [53]. This requires the development of Cas9 orthologs that not only recognize a variety of PAM sequences but also have a size compatible with the most commonly used delivery vector, AAV. 

Because with base editing there is the possibility to induce bystander edits, an ideal base editor should possess a narrow activity window allowing a focus solely on the target base. Nonetheless, such a narrow activity window would require the ability of the base editor to be deployable for a wide range of PAM sequences, raising an additional shortcoming of the base editing system [18]. Namely, for effective base editing, the PAM, of which the sequence is limited per Cas9 ortholog, must be properly located in order to keep the target base within the activity window, thereby drastically impeding the number of appropriate target sites [61]. Even though SpCas9 provides the least restrictive PAM availability among the commonly employed Cas9 variants, the necessity of a PAM sequence dramatically restricts the targetability of this editor to ~26% of known pathogenic single nucleotide polymorphisms (SNPs) [18]. To overcome this shortcoming, several research groups have established enhanced versions of base editors containing alternative PAM demands or less restrictive PAM compatibilities. Regrettably, these adjustments were shown to yield an increase in off-target editing [61], prolonging the need for improved base editors with greater PAM flexibility and an unchanged—or, even more ideally, reduced—number of off-target events. 

On the other hand, prime editors are capable of introducing point mutations far (>30 bp) from the nicking site, resulting in greater targeting flexibility than Cas9 nuclease-mediated HDR, and less restrictive PAM availability in contrast to other precision editing methods, including base editing. Therefore, although less efficient, prime editing could complement base editing, not only to avoid bystander off-target effects but also in case of the lack of an appropriately located PAM [19,21]. 

### 3.3. Cell Fitness Effects 

The DSBs induced by CRISPR/Cas9 often trigger apoptosis as opposed to the intended genome edit. This DNA-damage toxicity highlights a major safety issue for the application of DSB-inducing CRISPR/Cas9 therapy [65]. Apart from these technical constraints, the immunogenic toxicity that is possibly caused by CRISPR/Cas9 should also be considered. Exogenous genetic material within the cells can lead to the rejection of these elements by the body’s immune system [66]. This limitation was further substantiated by Charlesworth et al., who revealed that pre-existing antibodies against the most employed Cas9 orthologs, SpCas9 and SaCas9, were found in more than half of the humans in their study [67]. For this reason, it is vital to find other variants of Cas9 that are immunologically safe in case of repeated gene therapy. 

Immunotoxicity is typically lower for base and prime editors since, once optimized, these techniques solely require a single administration, though this needs to be further investigated and characterized [68]. Interestingly, less stringent immunologic and physical barriers are present in the developing fetus compared to postnatal stages, making in utero genome editing appealing for the treatment of genetic diseases diagnosed before birth. Moreover, an SpCas9 immune response was observed after postnatal, but not in utero, adenoviral delivery of an SpCas9-based CBE [69]. Accordingly, the presence of anti-SpCas9 antibodies was demonstrated in the serum of adult but not fetal ABE recipients [47]. 

Additionally, since prime editors necessitate an RT template, random complementary DNA could potentially be incorporated into the genome, causing further safety concerns [70].

### 3.4. Genome Editing in Post-Mitotic Tissues

Given that NHEJ is the favored repair mechanism in somatic cells, unpredictable insertions or deletions can occur after DSB induction. Specifically, HDR is very inefficient in cardiomyocytes, and the DSB-repair relies on error-prone NHEJ [58]. This risk is tolerable for the knockout of a gene, but not as much for the correction of a disease-causing mutation [71]. The editing efficiency of CRISPR/Cas9 was shown to be improved by inhibiting NHEJ [72]; however, this has not been done in heart tissue and remains to be studied. Moreover, genome editing efficiency with CRISPR/Cas9 seems to be lower in cardiac muscle compared to the liver, as shown also in cardiac-specific Cas9 transgenic mice, where editing efficiency in cardiac cells is still low [52]. Consequently, the application of CRISPR/Cas9 in cardiac tissue is generally not sufficiently advanced and requires further development. 

Considering that both base editing and prime editing are dependent on cellular mismatch repair mechanisms as opposed to recombinant repair mechanisms, such as HDR, these more recent methods represent an alternative strategy for genome editing in non-dividing cells, such as cardiomyocytes.

Finally, as the heart is a post-mitotic organ, the repair of DSBs induced by CRISPR/Cas9 rarely occurs via HDR. Besides using base editing or prime editing, which do not rely on HDR, an alternative solution might be homology-independent targeted integration (HITI). This allows the insertion of exogenous DNA into the genome of dividing or non-dividing cells, such as cardiomyocytes, in a fashion that relies on NHEJ-based ligation [73]. 

### 3.5. Delivery

For genome editing to exert the desired effect, the components associated with the utilized system need to be transported to the nucleus of the target cells. Therefore, the delivery of genome-editing systems is a vital process. To achieve this, the different components of the chosen genome editing system require a robust delivery tool that allows the genome-editing machinery to reach the target cells. Researchers have explored various options for this, including delivering DNA or mRNA encoding Cas9 that allows in situ production of the protein, as well as directly delivering the native form of Cas9. Due to the large size of Cas9 and the negative charge of the gRNA, the cell membrane will naturally block such molecules from entering [74]. To overcome these obstacles, numerous approaches have been explored, including viral and non-viral vectors (for a recent and complete overview, see [75]).

Since viruses have developed to become highly effective at invading cells and inserting exogenous cellular components inside the host cell, viral particles have been employed for the delivery of genome editing systems. The viral genes responsible for replication are removed and replaced by a transgene of interest to ensure that the virus can still enter the host cell but is no longer able to replicate itself. Several types of viruses, including adenovirus, lentivirus, retrovirus, and AAV have been modified for utilization in gene therapy applications [16]. 

The AAV is currently the most commonly used viral vector for the delivery of Cas9 and its associated components [74]. This vector realizes consistent transgene expression in a broad range of tissues, both mitotic and post-mitotic, including the heart [76]. Different AAV-based clinical trials have already been performed for several diseases, such as hemophilia A [77], hemophilia B [78], and achromatopsia [79]. Currently, as reported on clinicaltrials.gov, AAV is being applied in different studies on ischemic and non-ischemic forms of cardiomyopathy as well as chronic heart failure, highlighting the interest of the scientific community in this therapeutic approach [80]. A major limitation of AAVs is their packaging capacity. Generally, large cargoes—such as SpCas9, base editors, and prime editors, containing dCas9 together with their accessory proteins—exceed the ~4.4 kb carrying capacity limit of AAVs [81]. A strategy to bypass this shortcoming is by using a smaller Cas9 ortholog, such as SaCas9, or by utilizing a dual-AAV strategy split-intein editor. This latter strategy was employed with base editors by Levy et al. and displayed therapeutically relevant efficiencies in a mouse heart [82]. The editors were divided into an N-terminal and C-terminal half and fused to half of a fast-splicing split-intein. Subsequent co-infection of these AAV particles gave rise to protein splicing in trans, thereby reconstituting the base editors. This approach has also been successfully employed for CRISPR/Cas9 [44,83] and prime editing [84]. 

An additional challenge concerning viral delivery is its long-term expression. Ideally, genome editing tools should be transiently expressed to reduce the risk of off-target nuclease genotoxicity and possible immune responses against the bacterial-derived proteins [81]. In this regard, non-viral delivery presents a more transient expression in comparison with viral delivery, thus making the non-viral approach safer.

There are numerous delivery methods that do not utilize viral vectors to transport Cas9 across the cell membrane. These comprise physical methods to disrupt cellular barriers, nanoparticle-mediated delivery, and chemical alterations to circumvent cellular barriers. The most substantial benefits these bring about are the evasion of the packaging size restrictions associated with viral vectors [74], allowing transient nuclease activity, as well as enabling repeated administration of gene-therapy reagents, although with less efficiency than viral delivery methods [81,85]. 

Temporarily breaching the cellular membrane to facilitate the entry of Cas9 and other genome editing components into the cell is a common technique used for transfecting cells in vitro. The most extensively studied method is electroporation, in which molecules can enter the cell and, subsequently, the nucleus, by means of a temporary electrical pulse that is applied to the respective cell, thereby making it more porous and non-selective. This technique is also viable for cardiomyocytes and, therefore, for ex vivo therapeutic approaches in cardiovascular diseases. However, in vivo electroporation is more challenging due to the complicated choice of parameters and the risk of damaging tissues [85].

Next to this highly efficient method of delivery, microinjection into one-cell embryos is also able to achieve high efficiency. Micron-scale needles pierce the cell membrane and directly release the cargo. Given that this process needs to be performed on individual cells, it is solely feasible in embryonic stages [74]. 

The most advanced in vivo non-viral delivery method is by using solid lipid nanoparticles (SLNs). This FDA-approved strategy is an appealing option for delivering Cas9 RNPs due to its high efficiency and outstanding clinical track record [81]. In an aqueous solution, lipids spontaneously form nanoparticles, and by simple mixing, cargo can be encapsulated within [74]. This lipid carrier can protect the cargo from immunological responses or enzymatic breakdown. With this method, effective genome editing can theoretically be achieved in a single administration, thereby reducing toxicity and immunogenicity from repeated dosage. 

The promising results accomplished by non-viral vectors notwithstanding, these approaches are generally not very efficient for the genome editing treatment of cardiovascular diseases, and vectors such as AAVs remain the most suitable for targeting the heart [86]. (Rincon et al., 2015).

## 4. Conclusions and Future Directions

The field of genome editing is rapidly advancing, and the most recently developed techniques, CRISPR/Cas9, base editing, and prime editing, show great potential for future broad applications in different fields.

For genome editing systems to be applicable in the clinic, further understanding and advances are necessary. Firstly, the improvement of technical approaches is needed to increase target specificity and to minimize or eliminate off-target effects. In addition, as base editing and prime editing technologies are still in their infancy, these systems need to be further characterized and optimized to allow for their therapeutic application in vivo. Moreover, since the most commonly used Cas9 proteins, SpCas9 and SaCas9, are large in size, they present a major delivery challenge in terms of packaging into AAVs. Future research is necessary to discover smaller Cas9 orthologs or to reduce the size of the presently available Cas9 proteins. Alternatively, novel or optimized delivery methods, such as SLNs, can aid the further enhancement of genome editing systems and their application in vivo for therapeutic use.

An additional limitation that calls for advancement is the immunogenicity of CRISPR/Cas9 proteins. As a consequence of frequent pre-existing adaptive immune responses to SpCas9 and SaCas9, Cas9 orthologs to which humans have not yet been exposed ought to be identified to reduce the risk of immunological responses. 

Since the possibilities and applications for genome editing have dramatically grown in the past years, an analogous increase in concerns about the ethics of human genome editing has been observed—in particular when it comes to its clinical applications—highlighting the importance of setting up specific regulatory systems. Genome editing on somatic cells potentially avoids ethical issues surrounding the permanent editing of the germline and allows for the treatment of already-diseased subjects. However, only when all limitations associated with genome editing techniques are resolved will it then be considered safe to employ this system in vivo, preventing dangerous and unethical outcomes.

In the most optimal scenario, optimized genome editors show increased target specificity, a low or absent rate of off-target effects, a small size, flexible PAM availability, and easy accessibility. This would create the most optimized form of a genome editor, which has minimal associated limitations, is easily applicable, and greatly outweighs the potential risks so that it could be applied in the clinic and benefit the medical world. While ex vivo genome editing is most likely the first possible clinical application, for direct in vivo editing of post-mitotic tissues, such as the heart, genome editing is probably still far away.

Thus far, only a limited number of genome editing studies have been conducted in the cardiac field, indicating that the opportunities provided by these tools have not yet been fully explored.

The potential of genome editing is great, and should therefore be further researched to allow these systems to substantially benefit humankind and possibly treat diseases that were hitherto thought to be untreatable.
